# Exploring the underlying mechanisms of obesity and diabetes and the potential of Traditional Chinese Medicine: an overview of the literature

**DOI:** 10.3389/fendo.2023.1218880

**Published:** 2023-08-01

**Authors:** Yan-kun Chen, Ting-ting Liu, Farah Khameis Farag Teia, Meng-zhou Xie

**Affiliations:** ^1^ School of Chinese Medicine, Hunan University of Chinese Medicine, Changsha, China; ^2^ Hunan Engineering Technology Research Center for Medicinal and Functional Food, Hunan University of Chinese Medicine, Changsha, China; ^3^ Key Laboratory of TCM Heart and Lung Syndrome Differentiation and Medicated Diet and Dietotherapy, Hunan University of Chinese Medicine, Changsha, China; ^4^ Department of Agro-technology, Medicinal and Aromatic Plants and Traditional Medicine Research Institute, National Centre for Research, Khartoum, Sudan

**Keywords:** Traditional Chinese Medicine, diabetes, gut microbiota, obesity, literature review

## Abstract

Obesity and diabetes are closely related metabolic disorders that have become major public health concerns worldwide. Over the past few decades, numerous studies have explored the underlying mechanisms of these disorders and identified various risk factors, including genetics, lifestyle, and dietary habits. Traditional Chinese Medicine (TCM) has been increasingly recognized for its potential to manage obesity and diabetes. Weight loss is difficult to sustain, and several diabetic therapies, such as sulfonylureas, thiazolidinediones, and insulin, might make it harder to lose weight. While lifestyle changes should be the primary approach for people interested in lowering weight, drugs are also worth investigating. Since some of the newer glucose-lowering medications that cause weight loss, such as glucagon-like peptide-1 receptor agonists (GLP-1 RAs) and sodium-glucose cotransporter 2 inhibitors (SGLT2i), are additionally utilized or are under consideration for use as anti-obesity drugs, the frontier between glucose-lowering medication and weight loss drugs appears to be shifting. This review provides an overview of the literature on the underlying mechanisms of obesity and diabetes and the prospect of TCM in their management. We discuss the various TCM interventions, including acupuncture, herbal medicine, and dietary therapy, and their effects on metabolic health. We also highlight the potential of TCM in regulating gut microbiota, reducing inflammation, and improving insulin sensitivity. The findings suggest that TCM may provide a promising approach to preventing and managing obesity and diabetes. However, further well-designed studies are needed to confirm the efficacy and safety of TCM interventions and to elucidate their underlying mechanisms of action.

## 1 Introduction

**Figure 1:**
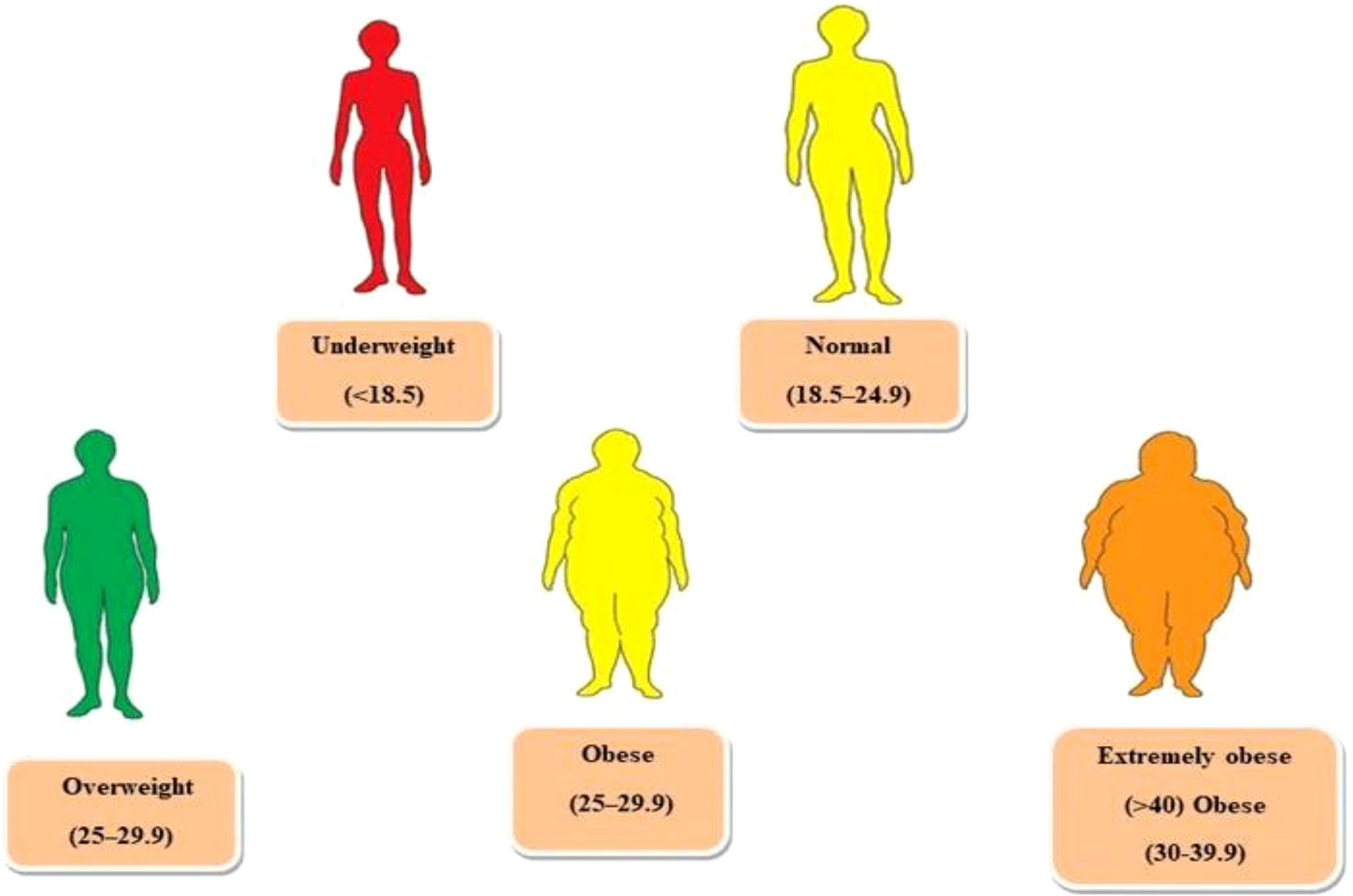
WHO recommended categorizing weight based on the body mass index (BMI).

Obesity is the accumulation of excess fat tissue in the body, which can occur at any age, and it is characterized by increased body and fat mass, hormone imbalances, eating patterns, and genetic factors. This condition has significantly contributed to the global burden of chronic diseases, including type 2 diabetes (T2D), cardiovascular disease, and asthma. It is considered a worldwide pandemic, and approximately 2.8 million people die from its complications annually (1). Directly measuring fat throughout the body is impossible, so the body mass index (BMI) is commonly used to evaluate the relationship between weight and height. Other methods, such as waist circumference, waist-to-hip ratio, skinfold thickness, and bio-impedance, are also utilized to assess overweight and obesity (2). If individuals fall into the heavy range of BMI, they are more likely to develop other diseases such as T2D, hypertension, cardiovascular diseases, and gallstones. The risk is moderate for those in obesity class 1, severe for those in obesity class 2, and very high for those with extreme obesity, especially if they already have other obesity-related diseases (3). **Figure 1** illustrates the BMI classification recommended by the World Health Organization and the National Institute of Health in the United States.

Visceral and subcutaneous are the two types of fats in the human body. Visceral fat is the one that gets deposited around organs such as the liver, pancreas, and kidneys, among others. This type of fat is also known as active fat because it significantly impacts hormonal activity. According to research, visceral fat accumulation can result in metabolic syndrome and insulin resistance, affecting appetite and body fat distribution (4). Subcutaneous fat, on the other hand, is the fat located beneath the skin and can be felt in areas like the underarms and legs. Fat distribution in the body can result in two types of shapes, apple, and pear. People with an apple shape tend to accumulate fat in the upper region of their waist, abdomen, neck, arms, and shoulders (5). This physique is predominantly linked with visceral fat, heightening the possibility of developing T2D.

**Figure 2:**
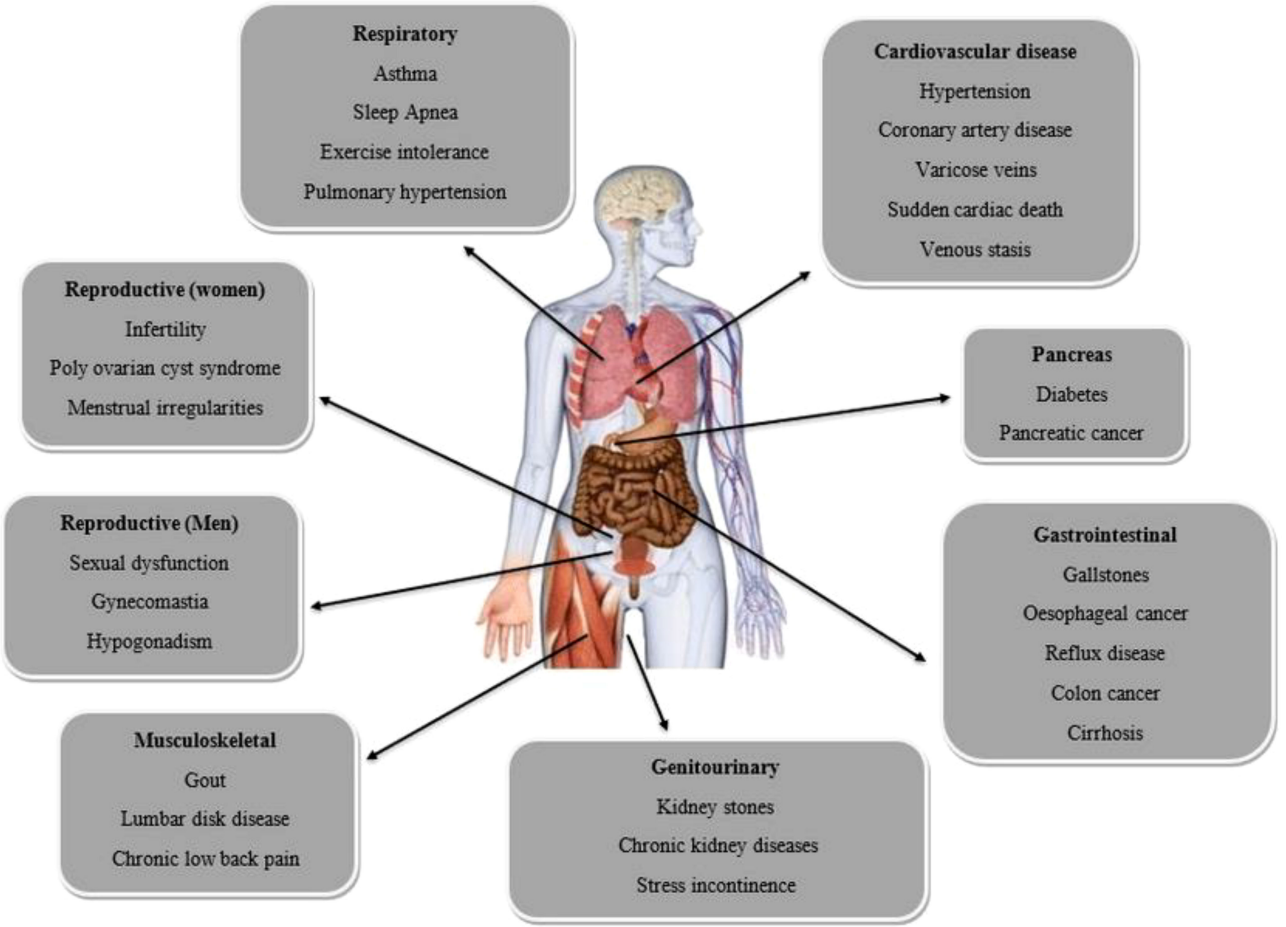
Obesity-related complications.

Conversely, individuals with a pear-shaped body have fat stored in their hips and thighs, resulting in lower visceral fat levels and a diminished risk of weight-related ailments (6). Obesity can give rise to numerous complications, such as reproductive problems for both genders, respiratory and cardiovascular illnesses, and issues with the gastrointestinal system and pancreas, as illustrated in (**Figure 2**). The United States ranked first in obesity, followed by China and India, according to the Organization for Economic Co-operation and Development (OECD) in 2017 (7). There has been a significant surge in obesity rates from 1999-2000 to 2015-2016 (8). In 2016, the World Health Organization (WHO) stated that of 1.9 billion overweight adults aged 18 and above, 650 million had obesity (2). An estimated 25 million individuals perish yearly due to obesity or being overweight (9, 10).

Moreover, a study by WHO in 2019 found that approximately 38.2 million children under 5 were either overweight or suffering from obesity (11). The individual’s lifestyle and dietary choices are crucial in contributing to the development of obesity (12). Foods that are high in fat and sugar tend to have low micronutrient content, which can lead to weight gain. Excessive intake of processed grains, unhealthy snacks, and sugary drinks may lead to a more excellent waist-to-hip ratio and heightened accumulation of body fat (13–16).

Diabetes is a chronic condition characterized by elevated blood sugar levels due to insufficient insulin production or the body’s inability to use insulin effectively. Insulin is a hormone produced by the pancreas that helps the body absorb and use glucose from food as energy (17). There are three primary types of diabetes: type 1, type 2, and gestational diabetes. Type 1 diabetes is an autoimmune disease that occurs when the immune system attacks and destroys the cells in the pancreas responsible for insulin production (18). This type of diabetes is typically diagnosed during childhood or adolescence and requires lifelong insulin therapy (19). T2D is the most common form, accounting for roughly 90% of cases, and occurs when the body becomes resistant to insulin’s effects. The pancreas can no longer produce enough insulin to meet demand (20). T2D is often linked to lifestyle factors such as obesity and physical inactivity but can be managed through diet, exercise, medication, or insulin therapy (21). Gestational diabetes develops during pregnancy and is caused by hormones that make it harder for the body to use insulin effectively. While this type of diabetes typically resolves after giving birth, women who develop gestational diabetes have an increased risk of developing T2D later in life, as do their children (22).

If diabetes is not managed correctly, it can result in various complications, such as cardiovascular disease, nerve damage, kidney disease, blindness, and amputations. Nevertheless, individuals with diabetes can maintain a long and healthy life with proper treatment and management, which may involve a blend of medication, lifestyle alterations, and frequent monitoring of blood glucose levels. This can aid in preventing the development of complications associated with diabetes (23, 24). The focus of this review is to explore the underlying mechanisms of obesity and diabetes and to evaluate the potential of Traditional Chinese Medicine (TCM) in managing these conditions. The study examined the current understanding of the pathophysiology of obesity and diabetes and investigated how TCM may help address these conditions. The aim is to provide a comprehensive and critical analysis of the existing research in this field and to assess the potential of TCM as a complementary or alternative treatment option for obesity and diabetes. The present study used a comprehensive search in major scientific databases, including PubMed, Scopus, and Web of Science, to identify relevant studies. The search used keywords related to obesity, diabetes, Traditional Chinese Medicine, underlying mechanisms, and their various synonyms. The search was limited to articles published between 2019 and 2023, with no language restrictions. Inclusion criteria encompassed original research articles, review articles, and meta-analyses investigating the relationship between obesity, diabetes, and TCM at the molecular, cellular, and clinical levels. Studies focusing on the mechanisms of action of TCM interventions, such as herbal remedies, acupuncture, and dietary modifications, were also included. Exclusion criteria consisted of studies that primarily focused on non-TCM interventions or those unrelated to the specific topic of interest.

## 2 The underlying mechanisms of obesity and diabetes

### 2.1 Insulin resistance

Insulin resistance refers to the decreased sensitivity of cells to insulin, which increases insulin levels in the bloodstream. It is considered a significant risk factor for obesity and is believed to be critical in its onset and advancement. The mechanisms by which insulin resistance mediates obesity are complex, but several key pathways have been identified (25). One of the primary mechanisms insulin resistance promotes obesity is its effects on regulating glucose metabolism. Insulin is an essential hormone for maintaining blood glucose levels within a healthy range. When insulin levels are high, glucose is taken up by cells and used for energy. However, in insulin-resistant individuals, cells become less responsive to insulin, leading to elevated glucose levels in the bloodstream (25, 26). The body produces more insulin to compensate, increasing insulin levels. These high insulin levels can promote the storage of excess glucose as fat, leading to weight gain and obesity. Another important mechanism by which insulin resistance promotes obesity is through its effects on the regulation of lipids. Insulin also regulates lipid metabolism; insulin-resistant individuals often have abnormal lipid profiles (27). For example, they may have elevated triglyceride levels, a type of fat in the blood. High triglyceride levels can promote fat storage in adipose tissue, leading to weight gain and obesity.

Insulin resistance can also promote obesity through its effects on regulating appetite and energy expenditure. Insulin regulates several hormones that control appetite and metabolism, including leptin, ghrelin, and adiponectin (28). Insulin-resistant individuals may have abnormal levels of these hormones, which can lead to increased appetite and reduced energy expenditure. This can lead to a positive energy balance, which promotes weight gain and obesity. Finally, insulin resistance can promote obesity through its effects on inflammation (29). Chronic low-grade inflammation is linked to insulin resistance, which may encourage the emergence of obesity and other metabolic disorders. Inflammatory cytokines produced by adipose tissue can impair insulin signaling and promote insulin resistance, further exacerbating the cycle of weight gain and metabolic dysfunction (30).

### 2.2 Inflammation

Obesity and diabetes are two closely related chronic diseases that are major health concerns worldwide. Research has shown that inflammation plays a crucial role in developing both conditions. When the immune system responds to infection or injury, inflammation occurs naturally. However, if it persists over a long period, chronic inflammation can result in various health issues, such as obesity and diabetes (31). One mechanism by which inflammation contributes to obesity and diabetes is releasing cytokines. Cytokines are signaling molecules secreted by immune cells and play a critical role in inflammation. In obese individuals, there is an increase in the production of cytokines such as tumor necrosis factor-alpha (TNF-α) and interleukin-6 (IL-6) by adipose tissue (31, 32). The cytokines found to hinder the function of insulin, which is crucial in regulating blood sugar levels, have been demonstrated to promote insulin resistance, a defining characteristic of T2D. Persistent inflammation associated with obesity plays a role in developing this disease. Furthermore, inflammation also triggers the activation of the nuclear factor kappa B (NF-κB) pathway, which is a transcription factor that regulates gene expression during inflammation. Studies indicate that activation of the NF-κB pathway occurs in the adipose tissue of obese individuals and contributes to insulin resistance (33, 34). Additionally, NF-κB pathway activation has been linked to the onset of T2D. Inflammation can also contribute to obesity and diabetes by altering the gut microbiome. The gut microbiome refers to the trillions of microorganisms that live in the human gut and play a critical role in maintaining health (35). Studies have shown that obesity and diabetes are associated with alterations in the gut microbiome, and inflammation may be a contributing factor. Inflammation can lead to changes in the composition of the gut microbiome, which can, in turn, contribute to metabolic dysfunction (36).

### 2.3 Hormonal imbalances

Obesity and diabetes are two of the most prevalent chronic diseases worldwide. Hormonal imbalances can contribute to the development and progression of both conditions, as they can affect the regulation of appetite, energy metabolism, and glucose homeostasis. The two main hormones involved in these processes are insulin and leptin. In this essay, we will examine how imbalances in insulin and leptin can lead to obesity and diabetes (37). Insulin is a hormone the pancreas produces that is critical in regulating glucose metabolism. The process of glucose uptake into cells and its subsequent storage as glycogen in the liver and muscles is enhanced by insulin (38). Obesity and T2D commonly display insulin resistance, characterized by a reduced responsiveness of the body’s cells to insulin. Consequently, the pancreas compensates by producing more insulin to maintain normal blood glucose levels. This can result in hyperinsulinemia, characterized by elevated insulin levels in the bloodstream (39). Hyperinsulinemia has been linked to obesity, as it facilitates fat storage in adipose tissue and impedes the breakdown of stored fat. It can also contribute to the development of diabetes by inhibiting glucose uptake into cells and promoting glucose production by the liver. Leptin is a hormone adipose tissue produces critical in regulating energy balance. It acts on the hypothalamus, a brain region controlling appetite and energy expenditure (40). Leptin signals the brain when energy stores are sufficient, reducing appetite and increasing energy expenditure. Leptin resistance is another common feature of obesity. It occurs when the brain becomes less responsive to leptin, and the body produces more leptin to compensate. This condition can lead to hyperleptinemia, characterized by high levels of leptin in the blood (40, 41). Hyperleptinemia can contribute to obesity by promoting fat storage in adipose tissue and inhibiting the breakdown of stored fat. Impaired glucose uptake into cells and increased glucose production by the liver, both of which can be caused by it, may also play a role in the development of diabetes (42).

### 2.4 Genetic predisposition

Obesity and diabetes are complex disorders resulting from genetic and environmental factors interplay. The genetic predisposition to these disorders has been extensively studied, and several mechanisms have been proposed to explain their mediation by genetics. One tool of obesity mediation by genetics is regulating appetite and energy expenditure. Several gene roles in appetite regulation have been identified, such as the leptin and melanocortin-4 receptor genes (43). Leptin is a hormone adipose tissue produces that regulates appetite and energy expenditure by acting on the hypothalamus. Mutations in the leptin gene or its receptor can lead to leptin resistance, resulting in increased appetite and reduced energy expenditure, leading to obesity (44). Similarly, mutations in the melanocortin-4 receptor gene can also increase hunger and obesity. Another mechanism of obesity mediation by genetics is regulating adipose tissue distribution. The distribution of fatty tissue in the body, particularly visceral adipose tissue, strongly predicts metabolic disorders such as diabetes and cardiovascular disease (45). Several genes that play a role in adipose tissue distribution have been identified, such as the FTO and PPARG genes. Variants in the FTO gene have been associated with increased body mass index and obesity. This effect is believed to be mediated by regulating adipose tissue distribution (45, 46). Similarly, variants in the PPARG gene have been associated with increased visceral adipose tissue, insulin resistance, and T2D. The genetics behind the development of diabetes are intricate and involve multiple factors. One way in which genetics contributes to diabetes is through the regulation of insulin secretion and sensitivity. Specific genes, such as the TCF7L2 gene and the insulin receptor gene, are involved in this process. Research has shown that variants in the TCF7L2 gene are strongly linked to a higher risk of T2D, which is believed to occur due to reduced insulin secretion (47). Similarly, mutations in the insulin receptor gene can lead to insulin resistance, leading to T2D. Genetics can also mediate diabetes by controlling the maintenance of glucose balance, which is another disease mechanism (48). Glucose homeostasis is tightly regulated by a complex interplay between several hormones, including insulin, glucagon, and amylin. Several gene roles in glucose homeostasis have been identified, such as the KCNJ11 gene and the HNF1A gene. Variants in the KCNJ11 gene have been associated with impaired insulin secretion and an increased risk of T2D. In contrast, mutations in the HNF1A gene can lead to impaired glucose homeostasis and maturity-onset diabetes of the young (49).

### 2.5 Lifestyle factors

Obesity and diabetes are two of the most prevalent chronic diseases worldwide, and their incidence is rising due to lifestyle factors. Obesity, characterized by excessive body fat accumulation, is a significant risk factor for T2D due to insulin resistance and impaired insulin secretion. Lifestyle factors such as sedentary behavior, unhealthy diet, and inadequate sleep are known to mediate the mechanisms of obesity and diabetes (50). Sedentary behavior, such as prolonged sitting or inactivity, has been linked to increased obesity and diabetes risk. Physical inactivity leads to decreased energy expenditure, reduced muscle mass, and impaired glucose metabolism, contributing to insulin resistance and diabetes development (51). Regular exercise can improve insulin sensitivity, glucose uptake, and body weight, reducing the risk of diabetes and obesity. An unhealthy diet, characterized by a high intake of refined carbohydrates, saturated and trans fats, and a low fiber intake of fruits and vegetables, is another crucial factor in developing obesity and diabetes (52). The high glycemic load of refined carbohydrates leads to rapid glucose absorption, causing insulin spikes and subsequent insulin resistance. The saturated and trans fats in unhealthy diets contribute to weight gain, insulin resistance, and inflammation, leading to obesity and diabetes. In contrast, a healthy diet that includes whole grains, fruits, vegetables, and moderate amounts of healthy fats, can help prevent and manage obesity and diabetes (53). Inadequate sleep, characterized by insufficient duration and poor quality, has been linked to increased obesity and diabetes risk. Sleep deprivation disrupts the regulation of hormones that control appetite and energy metabolism, leading to increased food intake, decreased physical activity, and altered glucose metabolism, contributing to obesity and diabetes (54). Adequate sleep, on the other hand, can improve insulin sensitivity, reduce appetite, and promote weight loss, reducing the risk of diabetes and obesity (55).

### 2.6 Gut microbiota

Obesity and diabetes are chronic metabolic disorders that affect a large portion of the global population. Recent research has highlighted the potential role of gut microbiota in developing and progressing these diseases. Gut microbiota refers to the trillions of microorganisms that inhabit the human gut, including bacteria, viruses, and fungi (56). These microorganisms regulate various metabolic processes, including energy homeostasis, glucose metabolism, and inflammation. One of the critical mechanisms by which gut microbiota mediates obesity is regulating energy balance. Gut bacteria have been shown to influence the amount of energy extracted from the diet by breaking down complex carbohydrates and other nutrients that are resistant to digestion by human enzymes (56, 57). This produces short-chain fatty acids (SCFAs), which the host can use as an energy source. However, excessive production of SCFAs can lead to increased fat storage and obesity. Another mechanism by which gut microbiota contributes to the development of obesity is through the regulation of appetite and satiety (58).

**Figure 3:**
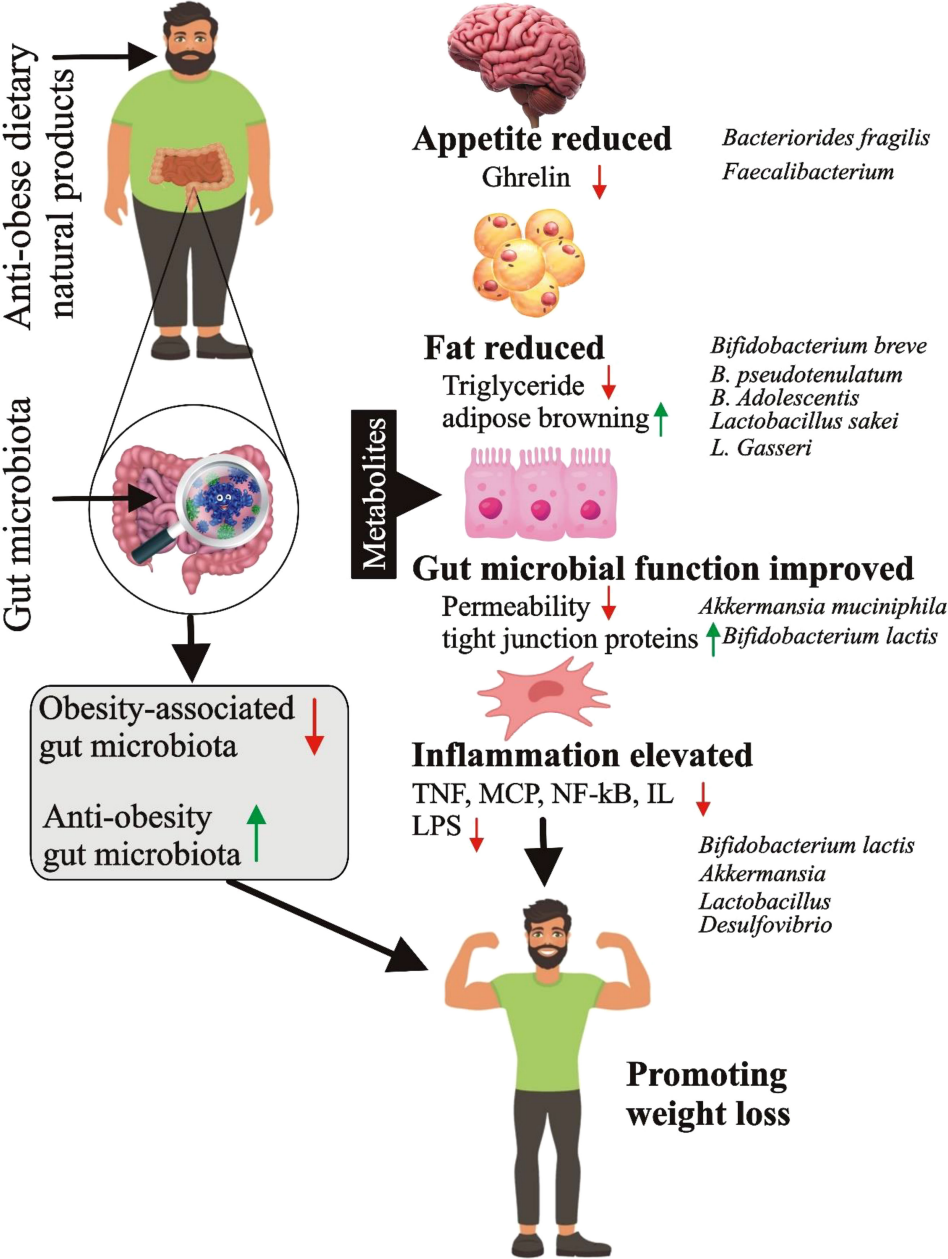
Mechanism of obesity mediation by gut microbiota (Figure credit: Meng-zhou Xie).

Studies have shown that gut bacteria, such as ghrelin, leptin, and serotonin, can produce various hormones and neurotransmitters that regulate hunger and food intake. Disruption of the regulation can cause an increase in food consumption and weight gain, as mentioned in reference (59). The gut microbiota, aside from being linked to obesity, has also been connected to diabetes development. The gut bacteria play a critical role in regulating glucose metabolism, one of the fundamental mechanisms contributing to diabetes development, as explained in reference (60). Various pathways have been discovered through which gut bacteria impact glucose metabolisms, such as the production of SCFAs that improve insulin sensitivity and the regulation of intestinal permeability that affects glucose absorption from the gut. Moreover, gut microbiota contributes to diabetes development through inflammation regulation, a hallmark of diabetes. Gut bacteria play a vital role in modulating the inflammatory response in the gut and systemic circulation (61, 62). Dysbiosis, or an imbalance in the gut microbiota, has been associated with increased pro-inflammatory cytokine levels that can cause insulin resistance and impaired glucose metabolism (63, 64) (**Figure 3**).

### 2.7 Environmental factors

Obesity and diabetes are complex conditions that various environmental factors can influence. These factors include dietary habits, physical activity, stress, pollution, and socioeconomic status. This response will explore how environmental factors can mediate obesity and diabetes. One primary ecological factor contributing to obesity and diabetes is dietary habits (65). The risk of developing obesity and diabetes can be heightened by consuming diets high in calories, fats, and sugars. Excess calories from these diets are stored as fat, leading to weight gain, and can also cause insulin resistance, which impairs glucose uptake by cells and can lead to the development of diabetes (66).

Moreover, a lack of nutrient-dense foods, such as fruits and vegetables, can lead to deficiencies in essential vitamins and minerals, further exacerbating the risk of obesity and diabetes (67). Another environmental factor that can contribute to obesity and diabetes is physical activity (65). Sedentary lifestyles, such as spending extended periods sitting, can reduce metabolic rate, decreasing calorie burning and weight gain. Additionally, physical inactivity can increase insulin resistance, making it more difficult for cells to use glucose effectively, thereby increasing the risk of diabetes. Conversely, regular physical activity can improve insulin sensitivity, aid weight management, and reduce the risk of developing both obesity and diabetes (68). Stress is another environmental factor that can contribute to obesity and diabetes. Chronic stress can lead to releasing cortisol, a hormone that increases appetite and can cause weight gain.

Moreover, cortisol can impair glucose uptake by cells, leading to insulin resistance and increasing the risk of diabetes. Therefore, effective stress management techniques, such as mindfulness, exercise, and social support, can help reduce the risk of developing obesity and diabetes (69, 70). Environmental factors such as pollution and socioeconomic status can also play a role in developing obesity and diabetes. Insulin resistance and the onset of diabetes have been associated with air pollution. In contrast, poverty, limited availability of healthy food choices, and unsafe physical activity environments can contribute to obesity and diabetes (71–73).

## 3 Role of Traditional Chinese Medicine (TCM)

Traditional Chinese Medicine (TCM) has a long history of use for treating various health conditions, including obesity and diabetes. TCM adopts a comprehensive perspective towards well-being, perceiving the body as an intricate network of interrelated components that both internal and external factors can influence. TCM uses a combination of modalities, including herbal medicine, acupuncture, dietary therapy, and lifestyle changes, to promote balance and harmony within the body and restore optimal health (73–75). Obesity is a growing health problem worldwide, and TCM has been used for centuries to address this condition. In TCM, obesity is seen as a result of imbalances within the body, such as dampness and phlegm accumulation, qi stagnation, and spleen and stomach deficiency (76). TCM practitioners will first evaluate the patient’s constitution and identify any underlying imbalances. Then, they will develop a personalized treatment plan that may include a combination of acupuncture, herbal medicine, and dietary therapy (77) (**Table 1**)

**Table 1:** Summary of Traditional Chinese medicine in treating obesity and diabetes.

Traditional Chinese Medicine	Medicine Formula	Function in Treating Obesity	Function in Treating Diabetes	References
*Rhizoma coptidis*	Huang Lian	Reduces body weight by inhibiting fat accumulation and improves insulin resistance	Lowers blood glucose levels by improving insulin resistance	(78)
*Semen cassiae*	Jue Ming Zi	Reduces body weight by promoting lipid metabolism and reducing lipid absorption	Lowers blood glucose levels by improving insulin sensitivity and promoting insulin secretion	(79)
*Fructus crataegi*	Shan Zha	Reduces body weight by reducing lipid accumulation and improving digestion	Enhancing insulin sensitivity and decreasing insulin resistance results in a decrease in blood glucose levels	(80)
*Radix puerariae*	Ge Gen	Reduces body weight by increasing energy expenditure and reducing fat accumulation	Enhancing insulin sensitivity and stimulating glucose uptake results in a reduction of blood glucose levels	(81)
*Folium mori*	Sang Ye	Reduces body weight by inhibiting fat accumulation and improving lipid metabolism	Enhances insulin sensitivity and encourages the secretion of insulin, resulting in a reduction of blood glucose levels	(82)
*Rhizoma polygonati*	Huang Jing	Reduces body weight by reducing fat accumulation and improving lipid metabolism	Enhancing insulin sensitivity and stimulating insulin secretion can result in a reduction of blood glucose levels.	(83)
*Radix astragali*	Huang Qi	Reduces body weight by increasing energy expenditure and reducing lipid accumulation	Enhances insulin sensitivity and facilitates glucose uptake, resulting in a reduction in blood glucose levels	(84)
*Radix ginseng*	Ren Shen	Reduces body weight by improving energy metabolism and reducing fat accumulation	Lowers blood glucose levels by improving insulin sensitivity and promoting insulin secretion	(85)

### 3.1 Acupuncture

For thousands of years, acupuncture has been a traditional Chinese medicine method to address various conditions, such as diabetes and obesity. This technique entails the insertion of thin needles into particular locations on the body, with the belief that it enhances the flow of energy or Qi, bringing about equilibrium within the body (86–88). In tackling obesity, acupuncture has shown promise in clinical studies by effectively decreasing body weight and body mass index (BMI). The needles are inserted into specific points on the body, including the ears, stomach, and spleen, which are thought to regulate appetite and metabolism. Acupuncture may also help to reduce inflammation in the body, which can contribute to weight gain and obesity-related health problems (89, 90).

Similarly, acupuncture can also be a helpful treatment for diabetes. By enhancing insulin sensitivity and decreasing insulin resistance, acupuncture has the potential to regulate blood sugar levels. The needles are typically inserted into points on the hands, feet, and ears, connected to the pancreas and other organs involved in blood sugar regulation (91). Furthermore, acupuncture can help to alleviate some of the symptoms of diabetes, such as neuropathy and nerve pain. Acupuncture has demonstrated the ability to trigger the production of endorphins, which are natural chemicals in the body that alleviate pain. This could potentially enhance diabetes management and decrease the likelihood of complications by minimizing discomfort and promoting overall health (92, 93). Acupuncture deforms connective tissue and increases the release of different molecules in acupoints as part of its anti-inflammatory impact, further activating the NF-κB, MAPK, and ERK pathways in mast cells, fibroblasts, keratinocytes, and monocytes/macrophages. Acupuncture-activated acupoints have somatic afferents that send sensory signals to the spinal cord, brainstem, and hypothalamus neurons. Acupuncture stimulates multiple neuro-immune pathways after information integration in the brain, such as the hypothalamus-pituitary-adrenal axis, which ultimately acts on immune cells via the release of critical neurotransmitters and hormones, the vagus-adrenal medulla-dopamine, the cholinergic anti-inflammatory, and sympathetic pathways (94–96).

### 3.2 Herbal medicine

Traditional Chinese Medicine (TCM) has been used for thousands of years to treat various health conditions, including obesity and diabetes. In TCM, obesity is viewed as a result of an imbalance in the body’s energy or “qi,” while diabetes is seen as a disorder of the body’s “yin” and “yang.” TCM practitioners use a variety of herbal medicines to address these imbalances and help promote weight loss and better blood sugar control (97, 98). One commonly used herb in TCM for treating obesity and diabetes is ginseng. Ginseng has been found to have anti-obesity and anti-diabetic effects, as it can help reduce insulin resistance, improve glucose metabolism, and increase energy expenditure (99).

Additionally, it has been shown to positively affect the gut microbiota, which can also contribute to weight loss (100). Another popular herb used in TCM for treating obesity and diabetes is bitter melon. Compounds present in bitter melon aid in regulating blood sugar levels and enhancing insulin sensitivity. It also has been found to have a mild appetite suppressant effect, which can aid in weight loss (101). Cinnamon is also commonly used in TCM for its anti-diabetic properties. It can help reduce fasting blood sugar levels, improve insulin sensitivity, and reduce inflammation. Cinnamon has also been shown to positively affect lipid metabolism, which can contribute to weight loss (102). In addition to these herbs, TCM practitioners may also recommend other lifestyle modifications, such as dietary and exercise, to help address obesity and diabetes. For example, TCM dietary guidelines often emphasize consuming nutrient-dense whole foods, such as vegetables, fruits, and whole grains, while limiting the intake of refined sugars and processed foods (103). In low-impact activities like tai chi and qigong, exercise can also help improve energy flow and promote overall health. While TCM may not cure obesity and diabetes, it can provide a valuable adjunct to conventional medical treatments. By addressing underlying imbalances in the body’s energy and promoting healthy lifestyle habits, TCM can help patients achieve better weight management and blood sugar control. As always, anyone considering herbal medicines should consult a qualified TCM practitioner or medical professional to ensure the treatment is safe and appropriate for their needs (104).

### 3.3 Dietary therapy

For centuries, Traditional Chinese Medicine (TCM) has relied on dietary therapy to address various health conditions, such as obesity and diabetes. TCM views the body as a whole and focuses on restoring balance and harmony between different organ systems. In TCM, obesity and diabetes are seen as imbalances in the body’s energy, or Qi, and can be treated through changes in diet and lifestyle (105, 106). In TCM, obesity is often associated with excessive dampness and phlegm in the body, which an unhealthy diet and lack of exercise can cause. The dietary therapy for obesity in TCM involves reducing the intake of fatty, greasy, and sweet foods while increasing the consumption of cooling foods that can help disperse dampness, such as bitter melon, lotus leaf, and green tea. Eating smaller, more frequent meals is also recommended, and avoiding eating late at night (107). Regular exercise, especially low-impact activities like walking and tai chi, is also recommended to help increase circulation and burn fat. Diabetes, on the other hand, is seen as a deficiency of Qi and Yin in the body. Qi refers to the body’s energy, while Yin refers to the body’s moisture and nourishment. In TCM, diabetes is often treated with dietary therapy, acupuncture, and herbal medicine (108). The dietary treatment for diabetes in TCM involves reducing the intake of sweet and greasy foods while increasing the consumption of foods rich in Qi and Yin, such as yams, sweet potatoes, and lotus seeds. Eating smaller, more frequent meals and avoiding raw or cold foods are also recommended. Regular exercises, such as brisk walking or cycling, are also advised to help improve circulation and regulate blood sugar levels (109). Overall, TCM dietary therapy for obesity and diabetes focuses on achieving balance and harmony in the body rather than simply treating the condition’s symptoms. By making healthy nutritional and lifestyle choices and receiving acupuncture and herbal medicine treatments, individuals can help restore their body’s natural balance and improve their overall health and well-being (110).

### 3.4 Qi gong and tai chi

Qi Gong and Tai Chi are traditional Chinese practices that can be used as alternative therapies for treating obesity and diabetes. These practices are based on the principles of Traditional Chinese Medicine, which views the body as a complex system of interdependent parts that must be harmonious for optimal health. Qi Gong and Tai Chi are gentle exercises incorporating breathing techniques, mindfulness, and gentle movements to improve overall health and well-being (111–114). Obesity is a primary global public health concern linked to many health complications, such as diabetes. Qi Gong and Tai Chi are effective in helping individuals manage their weight and reduce the risk of developing obesity-related (115). These practices promote weight loss by improving metabolism, reducing stress, and increasing physical activity. One of the primary ways that Qi Gong and Tai Chi help with obesity is through stress reduction. Chronic stress significantly contributes to weight gain, as it can cause hormonal imbalances that increase appetite and decrease metabolism (116). Qi Gong and Tai Chi help to reduce stress by promoting relaxation, improving sleep quality, and reducing anxiety and depression.

Additionally, Qi Gong and Tai Chi are low-impact exercises that individuals of all fitness levels can practice. These practices can improve cardiovascular health, muscle strength, and flexibility, contributing to weight loss and overall health. Diabetes is a metabolic disorder that affects millions of people worldwide. While there is no cure for diabetes, lifestyle changes, including exercise, can help manage the disease. Qi Gong and Tai Chi effectively manage diabetes by controlling blood glucose, reducing inflammation, and improving cardiovascular health (117–120). One of the primary ways that Qi Gong and Tai Chi help manage diabetes is by improving blood glucose control. These techniques aid in managing blood sugar levels by enhancing insulin sensitivity and diminishing insulin resistance.

Moreover, the practice of Qi Gong and Tai Chi can potentially decrease inflammation, which is crucial in the onset and advancement of diabetes (121). Qi Gong and Tai Chi are effective traditional Chinese medicine options for managing obesity and diabetes. These practices promote overall health and well-being by reducing stress, improving physical activity levels, and helping to manage chronic diseases. Additionally, Qi Gong and Tai Chi are safe and gentle exercises that individuals of all ages and fitness levels can practice (122).

### 3.5 Massage and bodywork

Massage and bodywork therapies are integral to Traditional Chinese Medicine (TCM), a holistic healthcare system that focuses on restoring the balance of the body’s vital energy or Qi. TCM offers a range of therapeutic options for treating various health conditions, including obesity and diabetes (123). Obesity is a metabolic disorder caused by an imbalance between energy intake and expenditure, leading to excessive accumulation of body fat (124). TCM views obesity as a result of Qi stagnation, dampness accumulation, and spleen and stomach weakness. Massage and bodywork therapies can help to regulate Qi flow, improve digestion, and promote lymphatic drainage, which can help to reduce body fat and improve metabolic function. Some typical massage and bodywork techniques used in TCM for obesity include acupressure, cupping, and Guasha (125). Acupressure involves applying pressure to specific points on the body, called acupoints, which correspond to different organs and systems. By stimulating these points, acupressure can help to regulate the function of the spleen and stomach, promote digestion, and reduce food cravings. Cupping is a technique in which cups are placed on the skin to create a suction effect (126). This can help to stimulate blood flow and lymphatic drainage, which can help to eliminate excess fluids and toxins from the body. Gua sha involves using a smooth-edged tool to scrape the skin, which can help to improve circulation, reduce inflammation, and promote healing (127).

Diabetes is a condition affecting metabolism marked by elevated blood sugar levels, which can result in various complications such as nerve damage, kidney disease, and cardiovascular disease (128). TCM views diabetes as a result of Qi deficiency, Yin deficiency, and dampness accumulation. Massage and bodywork therapies can help to tonify Qi and Yin, regulate blood sugar levels, and improve circulation (125). Some standard massage and bodywork techniques used in TCM for diabetes include acupressure, moxibustion, and foot reflexology (129). Moxibustion involves burning a herb called mugwort over specific acupoints, which can help to notify Qi and improve circulation. Foot reflexology is the practice of exerting pressure on particular points on the feet that correspond to various organs and systems within the body. By stimulating these points, foot reflexology can assist in regulating blood sugar levels and enhancing overall well-being (130–133).

### 3.6 Tauroursodeoxycholic acid (TUDCA)

A naturally occurring hydrophilic bile acid called tauroursodeoxycholic acid (TUDCA) has been used for generations in CM. In chemical terminology, TUDCA is a taurine conjugate of ursodeoxycholic acid (UDCA). This drug has been accepted by the Food and Drug Administration (FDA) for use in treating primary biliary cholangitis in modern pharmacology (134). Recent research studies indicate that TUDCA’s functioning mechanisms extend beyond hepatobiliary conditions. Due to its cytoprotective effect, TUDCA has been demonstrated to have potential therapeutic applications in various disease models, including neurodegenerative diseases, obesity, and diabetes. TUDCA was identified as a chemical chaperone due to the mechanisms underlying its cytoprotective action, mostly associated with regulating the unfolded protein response (UPR) and reducing ER stress. In addition, TUDCA has been shown to reduce oxidative stress, control apoptosis and reduce inflammation in numerous *in-vitro* and *in-vivo* models of different diseases (135, 136).

### 3.7 Therapeutic effects of western medicine

The weight-related effects of drugs used to treat T2D vary; some show a beneficial effect on weight loss, some have weight-neutral effects, and some result in a gain in weight. Examining the currently available drug profile is crucial when weight loss is a priority to identify prospective areas for improving blood-glucose control and weight management. **Table 2** discusses several classes of drugs, including metformin, SGLT2 inhibitors, and GLP-1 agonists, and how they affect weight loss in individuals with T2D (137, 138).

**Table 2:** Summary of diabetes pharmacological treatments and their effect on weight loss.

Medication/Drug	Dose	Change in weight (kg)	Negative effects
*SGLT2 inhibitors			
Canagliflozin	100 to 300 mg daily/orally	~1 to 2 kg weight loss	Risk of amputation Risk of bone fracture Increased LDL level Risk of volume hypotension Genitourinary infections risk
Ertugliflozin	5 to 15 mg daily/orally		
Dapagliflozin	5 to 15 mm daily/orally		
Empagliflozin	10-25 mg daily/orally		
Metformin	500-100 mg (IR; tablets)/twice a day) 2000 mg (ER; tablets/daily)	~1 to 8 kg weight loss	Lactic acidosis B12 deficiency Diarrhoea, nausea, vomiting
*GLP1 Agonists			
Lixisenatide	10-20 mcg SC daily	~3 to 10 kg weight loss	Risk of thyroid C-cell tumor Risk of pancreatitis Injection site reactions Vomiting, nausea
Dulaglutide	0.75-1.5 mg SC weekly		
Exenatide	ER: 2 mg SC weekly IR: 5-10 mcg SC twice daily		
Semaglutide	Tablet: 3-14 mg daily Injection: 0.25-2.4 mg SC weekly		
Liraglutide	0.6-1.8 mg Titrate daily 0.6 mg SC daily		

*SGLT2; sodium-glucose cotransporter 2, GLP-1; glucose-like peptide-1, IR; immediate release, ER; extended release.

## 4 Hormone associated with obesity

### 4.1 Insulin

Insulin is an essential hormone responsible for regulating blood sugar levels within the body. After we consume food, carbohydrates are broken down into glucose, which is absorbed into the bloodstream. The pancreas produces insulin, which facilitates the transportation of glucose from the bloodstream to cells, where it can be utilized as energy or stored for future use (139). However, in individuals with obesity, their bodies may become less responsive to the effects of insulin, leading to elevated glucose levels in the bloodstream and an augmented likelihood of developing T2D (140). One of the main ways that insulin resistance contributes to obesity is through its effects on fat cells. Insulin helps to regulate the storage and breakdown of fat in the body. When insulin levels are high, fat cells store glucose as fat. When insulin levels are low, fat cells break down stored fat to release energy. However, in people with insulin resistance, fat cells become less responsive to insulin and are less able to take up glucose and store fat. As a result, more glucose remains in the bloodstream, leading to higher insulin levels and increased fat storage (141).

Another way that insulin resistance can contribute to obesity is through its effects on appetite and metabolism (142). Insulin helps regulate hunger and satiety by signaling to the brain that the body has enough to eat. When insulin levels are high, the brain receives signals to stop eating and start using stored energy. However, the brain may become less responsive to these signals in people with insulin resistance, leading to increased appetite and overeating (143). Insulin resistance can also affect the body’s metabolism or the rate at which it burns calories. When insulin levels are high, the body tends to store energy in the form of fat. However, in people with insulin resistance, the body may be less able to use stored fat for energy and instead rely on glucose as a fuel source. This can lead to lower metabolic rates and decreased calorie burning, challenging losing and maintaining a healthy weight (144).

### 4.2 Omentin

Omentin is a hormone that is primarily produced by adipose tissue, which is the tissue that stores fat in the body. It belongs to a group of hormones known as adipokines, which regulate metabolism and inflammation. Omentin is associated with obesity and metabolic disorders, and its levels in the body are altered in individuals with these conditions (145). Research has shown that omentin is essential in regulating insulin sensitivity, which is the body’s ability to respond to insulin and use glucose for energy. Insulin resistance, which is the impaired ability of cells to respond to insulin, is a common feature of obesity and is a risk factor for T2D (146). Omentin has been found to improve insulin sensitivity in animal studies, and lower levels of omentin have been observed in individuals with insulin resistance. In addition to its role in insulin sensitivity, omentin regulates inflammation in the body (147). Inflammation is a natural response of the immune system to injury or infection, but chronic inflammation is associated with various health conditions, including obesity, diabetes, and cardiovascular disease. Research has indicated that omentin possesses anti-inflammatory properties, and evidence suggests that heightened inflammation within the body is associated with lower levels of omentin (148). An interesting finding is that omentin levels seem to be influenced by the location of adipose tissue. Specifically, subcutaneous adipose tissue, located just beneath the skin, has been observed to produce greater amounts of omentin compared to visceral adipose tissue surrounding internal organs. This implies that how body fat is distributed could impact omentin levels and their impact on metabolism (149).

### 4.3 Leptin

Leptin, a hormone synthesized by adipose tissue or fat cells, significantly regulates body weight and metabolism. The amount of leptin released into the bloodstream is directly proportional to the body’s fat stores. Its primary function is communicating with the brain, inducing decreased appetite and increased energy expenditure (150). In people with obesity, there is often a condition called leptin resistance, in which the body becomes less responsive to the effects of leptin. This can lead to a cycle of overeating and weight gain, as the brain doesn’t receive the signal to decrease appetite or increase energy expenditure. Leptin resistance is thought to develop due to chronic overeating and high circulating leptin levels over an extended period. This leads to a desensitization of the receptors that respond to the hormone (151). Interestingly, while leptin resistance is commonly associated with obesity, not all people with obesity have leptin resistance, and not all people with leptin resistance are obese. Evidence suggests that other factors, such as genetics, inflammation, and environmental toxins, may play a role in developing leptin resistance (152, 153). In addition to regulating appetite and energy expenditure, leptin has other bodily functions, such as immune function, fertility, and bone metabolism. Therefore, disruptions in leptin signaling can have far-reaching effects on overall health and well-being (154).

### 4.4 Acylation stimulating protein (ASP)

ASP is a hormone found to regulate energy metabolism and adipose tissue physiology. ASP is produced primarily by adipocytes and is secreted into the circulation in response to food intake, especially dietary fat. Once in the bloodstream, ASP binds to its receptor, C5L2, expressed in adipocytes, muscle cells, and other tissues, and initiates various cellular responses (155). One of the main functions of ASP is to promote the uptake and storage of dietary fat in adipose tissue. ASP can encourage the production of fatty acids, or lipogenesis, and increase the absorption of fatty acids by adipocytes. This can lead to the enlargement of adipose tissue depots and potentially contribute to the development of obesity (156). Research has indicated that obese individuals have higher levels of ASP in their bloodstream than those who are lean. Additionally, ASP has been linked to glucose homeostasis and insulin sensitivity regulation. Some studies have demonstrated that ASP can facilitate glucose uptake in muscle cells and adipocytes and improve insulin sensitivity in these tissues (157). However, the impact of ASP on glucose metabolism appears to depend on the circumstances, as some studies suggest that ASP may hinder glucose tolerance and contribute to insulin resistance. The precise mechanisms underlying the effects of ASP on energy metabolism and glucose homeostasis are not fully understood (158). It is thought that ASP may act in concert with other hormones and signaling pathways, such as insulin and adipokine leptin, to regulate these processes. Evidence suggests that ASP may directly affect the hypothalamus, a brain region crucial in regulating energy balance (159).

The processes by which ASP increases triacylglycerol production are now firmly established. Stimulating the last (and most likely rate-limiting) enzyme involved in triacylglycerol production, diacylglycerol acyltransferase, has two distinct implications. The second is an increase in glucose-specific membrane transport. Increased diacylglycerol acyltransferase (EC 2.3.1.20) activity increases fatty acid incorporation into triacylglycerol and, as a result, adipocytes’ rate of fatty acid intake. The increase in specific membrane glucose transport, an additional effect of ASP, is also of considerable significance. In human skin fibroblasts, human adipocytes, and L6 myotubes, ASP increases glucose transport (160).

### 4.5 Ghrelin

Ghrelin is a hormone produced mainly by the stomach, although small amounts are also secreted by other organs such as the pancreas and small intestine (161). It stimulates appetite and promotes weight gain, making it an essential hormone in regulating energy balance and body weight. Ghrelin acts on the hypothalamus, a region in the brain that controls food intake and energy expenditure, as well as other areas involved in reward and motivation. The release of ghrelin is influenced by various factors such as fasting, stress, and sleep deprivation (162). It is secreted in higher amounts during fasting or calorie restriction periods, which may explain why people often experience intense hunger. Ghrelin levels also increase in stress response, which may contribute to overeating and weight gain in some individuals who use food as a coping mechanism (163).

Lack of sleep has also increased ghrelin levels, possibly contributing to the link between sleep deprivation and obesity (164). Ghrelin is believed to promote weight gain by several mechanisms. First, it increases appetite and food intake, increasing energy surplus and weight gain. Second, it slows down metabolism and reduces energy expenditure, which can also contribute to weight gain. Third, it promotes fat accumulation in adipose tissue by stimulating the release of growth hormones, insulin, and other hormones involved in fat storage (165). Studies have shown that ghrelin levels are often higher in obese individuals than those of average weight. This suggests that ghrelin may play a role in developing obesity and related metabolic disorders (166). However, the relationship between ghrelin and obesity is complex, and further research is needed to understand its role in this context entirely (167).

### 4.6 Peptides YY (PYY)

The endocrine cells in the gastrointestinal tract’s ileum and colon secrete a hormone called Peptide YY (PYY), which plays a vital role in controlling appetite and satiety by being released after food intake. PYY belongs to the pancreatic polypeptide family and is structurally similar to neuropeptide Y (NPY) and peptide YY2 (PYY2) (168). Several studies have shown that PYY levels are altered in individuals with obesity. In particular, it has been observed that obese individuals have lower levels of PYY compared to normal-weight individuals. This suggests that PYY may be involved in the pathophysiology of obesity (169). The exact mechanisms through which PYY regulates body weight are still not fully understood. One of the main ways PYY influences appetite and food intake is by acting on the hypothalamus, which is part of the brain that regulates energy balance. PYY activates neurons in the hypothalamus that suppress appetite and promote satiety (21). This leads to a reduction in food intake and an increase in feelings of fullness. In addition to its effects on appetite, PYY has also been shown to influence energy expenditure. Studies have demonstrated that PYY can increase energy expenditure by stimulating the sympathetic nervous system, which regulates metabolic processes such as thermogenesis and lipolysis (170). This suggests that PYY may be involved in regulating body weight through its effects on both food intake and energy expenditure. The role of PYY in treating obesity has been investigated in several clinical studies. One approach involves using PYY analogs, synthetic molecules that mimic the effects of endogenous PYY. Studies have demonstrated that these analogs can decrease food consumption and facilitate weight loss in both human subjects and animal models (171).

## 5 Recently developed treatments for obesity

Obesity is a chronic disease characterized by an excessive accumulation of body fat, which can lead to a range of health problems, including diabetes, heart disease, and stroke. While diet and exercise are the primary means of managing obesity, several recently developed treatment options can help individuals lose weight and maintain a healthy lifestyle.

### 5.1 Bariatric surgery

Bariatric surgery is a weight loss surgery that involves altering the digestive system to help individuals struggling with obesity loses weight. Obesity is a chronic condition affecting millions worldwide and can lead to various health complications such as heart disease, T2D, and sleep apnea (172). Bariatric surgery is often recommended for individuals with a body mass index (BMI) of 40 or higher or those with a BMI of 35 or higher with at least one obesity-related medical condition. There are different bariatric surgery procedures, each with its benefits and risks. The most common types include gastric bypass, sleeve gastrectomy, adjustable gastric banding, and biliopancreatic diversion with a duodenal switch (173). In gastric bypass surgery, the surgeon creates a small stomach pouch and reroutes the small intestine, limiting the amount of food consumed and absorbed by the body. Sleeve gastrectomy involves removing a portion of the stomach to reduce its size. In contrast, adjustable gastric banding involves placing an inflatable band around the top part of the stomach to restrict food intake (174). Bariatric surgery is an effective procedure that requires careful consideration and preparation. Before surgery, patients undergo a comprehensive evaluation to determine their suitability for the process and identify any underlying medical conditions that may affect the outcome (175). Patients must also undergo extensive counseling and education to help them understand the procedure’s risks and benefits and prepare them for the changes they must make to their lifestyle after the surgery. Bariatric surgery is not a magic solution to weight loss (176). While the surgery can help individuals lose significant weight, it requires a commitment to long-term lifestyle changes, including healthy eating habits and regular exercise. Patients undergoing bariatric surgery must also be monitored closely by their healthcare provider to ensure they meet their weight loss goals and address complications (177). The desire to participate in hormonal changes following bariatric surgery arises from two fundamental observations: (a) the weight loss seems to arise from reductions in appetite and food intake, implying that the surgical procedure interferes with the normal regulation of appetite and food intake, and (b) the reversal of T2D occurs a few days after surgery before any significant weight loss has occurred, implying that mechanisms other than weight loss are involved (178).

### 5.2 Endoscopic sleeve gastroplasty (ESG)

Endoscopic sleeve gastroplasty (ESG) is a relatively new, minimally invasive procedure for weight loss in people with obesity. The process involves using an endoscope, a thin tube with a camera, and surgical instruments attached to it to reduce the stomach size by creating a sleeve-like shape (179). This limits the amount of food the stomach can hold, leading to a feeling of fullness and reduced hunger. The procedure is usually done on an outpatient basis, and patients are given general anesthesia. Once the patient is sedated, the endoscope is inserted into the mouth and down the throat to reach the stomach. The surgeon gathers and folds the stomach tissue using sutures into a narrow tube shape, creating a sleeve-like structure (180). The sutures are then tightened to hold the sleeve in place. ESG typically takes 60 to 90 minutes, and patients are usually discharged on the same day. Most patients can return to normal activities within a few days after the procedure, although a liquid diet is generally recommended for the first week or so. ESG is effective for weight loss in people with obesity (181). Studies have shown that patients typically lose between 15% and 20% of their excess body weight within 12 months after the procedure. ESG has also improved various health markers, including blood pressure, cholesterol levels, and blood sugar control. However, ESG is not without risks. Complications can occur, although they are rare. These can include bleeding, infection, and perforation of the stomach or esophagus. Patients may also experience nausea, vomiting, and abdominal pain in the first few days after the procedure. ESG is not appropriate for everyone with obesity (182). It is generally recommended for people with a body mass index (BMI) between 30 and 40 who cannot lose weight through diet and exercise alone. People with certain medical conditions, such as inflammatory bowel disease or previous surgeries on the stomach or intestines, may not be candidates for ESG (183).

### 5.3 Duodenal-jejunal bypasses liner

The duodenal-jejunal bypass liner (DJBL) is a non-surgical weight loss treatment for obesity that involves the insertion of a temporary liner into the small intestine. The liner works by restricting the absorption of nutrients from food, leading to a reduction in calorie intake and weight loss. During the DJBL procedure, a flexible tube with a balloon at one end is inserted through the mouth and into the small intestine (184). Once in place, the balloon is inflated, creating a barrier that prevents food from coming into contact with the first part of the small intestine, called the duodenum. By bypassing the duodenum and restricting nutrient absorption, the DJBL promotes weight loss and helps improve metabolic conditions such as diabetes and high blood pressure (185). The DJBL is a reversible procedure and can be removed after six months. During this time, patients are advised to follow a structured diet and exercise program to maximize weight loss results. The DJBL is intended for patients with a body mass index (BMI) of 30 or higher who cannot lose weight through diet and exercise alone. Studies have shown that the DJBL can be an effective weight loss tool, with patients losing an average of 20-25% of their excess body weight during the six-month treatment period (186).

Additionally, the DJBL has been shown to improve metabolic conditions such as diabetes and high blood pressure, with some patients experiencing remission. Like any medical procedure, the DJBL does carry some risks, including nausea, vomiting, and abdominal pain. In rare cases, the DJBL can lead to more severe complications such as bowel obstruction, bleeding, or perforation. Patients considering the DJBL should discuss the risks and benefits with their healthcare provider to determine if it is the proper weight loss treatment for them (187).

### 5.4 GLP-1 receptor agonists

GLP-1 receptor agonists are primarily used to treat T2D but have also shown efficacy in managing obesity. These medications imitate the effects of GLP-1, a gut hormone that helps regulate appetite and blood glucose levels, resulting in a decrease in hunger and an increase in fullness, leading to reduced food intake and subsequent weight loss (188). Furthermore, they offer additional benefits by improving glycemic control and decreasing cardiovascular risk factors in individuals with obesity and T2D (189). One of the most commonly used GLP-1 receptor agonists for treating obesity is liraglutide, administered once daily by subcutaneous injection, leading to an average weight loss of 5-10% of initial body weight (185). Semaglutide, another GLP-1 receptor agonist administered once weekly by subcutaneous injection, is even more effective, resulting in an average weight loss of 15-20% of initial body weight (190). However, GLP-1 receptor agonists can cause side effects, including nausea, vomiting, and diarrhea, which can be minimized by starting with a low dose and gradually titrating. Additionally, they may increase the risk of pancreatitis and thyroid tumors, but the overall risk is low (191).

Semaglutide is a medication recently approved by the US Food and Drug Administration (FDA) for treating obesity in adults. It is a glucagon-like peptide-1 (GLP-1) receptor agonist, miming the action of a naturally occurring hormone called GLP-1 (185). The intestine releases GLP-1 in reaction to food consumption, and it triggers the pancreas to secrete insulin, thus aiding in regulating blood sugar levels. Semaglutide has been found to have a dual action of regulating blood sugar levels and inducing weight loss. Semaglutide works by activating the GLP-1 receptor in the brain, which results in decreased appetite and increased feelings of fullness or satiety (192). This leads to a reduction in food intake, resulting in weight loss. Moreover, it has been demonstrated that semaglutide decelerates the process of gastric emptying, which refers to the speed at which food exits the stomach and moves into the small intestine. This prolongs the feeling of fullness, which helps to reduce calorie intake and promote weight loss (193).

In clinical trials, semaglutide is effective in promoting weight loss in adults with a body mass index (BMI) of 30 or higher, which is considered obese, as well as those with a BMI of 27 or higher who have at least one weight-related health condition, such as T2D or high blood pressure (176). In one study, participants who received a once-weekly injection of semaglutide lost an average of 15% of their body weight over 68 weeks, compared to a 2.4% weight loss in the placebo group. Semaglutide is typically administered once a week via subcutaneous injection. The recommended starting dose is 0.25 mg per week, gradually increasing to 2.4 mg per week over 16 weeks (194). The medication should be combined with a reduced-calorie diet and increased physical activity for optimal results. Like any medication, semaglutide may cause side effects. The most common side effects reported in clinical trials include nausea, diarrhea, vomiting, and constipation. In rare cases, semaglutide may cause inflammation of the pancreas, which can be severe and requires immediate medical attention (195).

### 5.6 Digital health interventions

Digital health interventions use digital technologies to support and improve health outcomes. In the case of obesity treatment, digital health interventions can be an effective tool to help individuals achieve and maintain a healthy weight. These interventions can include a range of technologies, such as mobile apps, wearable devices, online programs, and virtual coaching (180). One of the key benefits of digital health interventions for obesity treatment is their ability to provide personalized support and feedback. Many digital health programs use algorithms to track an individual’s progress, provide personalized feedback, and adjust their plan accordingly. For example, a digital health app may use data on an individual’s weight, physical activity, and dietary habits to provide personalized recommendations on achieving their weight loss goals (196). Another advantage of digital health interventions is their accessibility. Individuals can access digital health programs from anywhere, which can be particularly beneficial for individuals with busy schedules or limited access to traditional healthcare resources. Additionally, digital health interventions can often be more cost-effective than conventional obesity treatments, making them more accessible to a broader range of individuals. Several studies have demonstrated the effectiveness of digital health interventions for obesity treatment (197). A systematic review of 23 randomized controlled trials found that digital health interventions were associated with significant body weight, BMI, and waist circumference reductions. Additionally, individuals who used digital health interventions reported high satisfaction with the programs. Despite the potential benefits of digital health interventions for obesity treatment, some challenges are associated with these programs (198). For example, some individuals may struggle to engage with the technology or find the programs overwhelming. Additionally, digital health interventions may not be appropriate for individuals with complex medical needs or require more intensive treatment (199).

## 6 Conclusion

Obesity and diabetes are complex metabolic disorders with multifactorial causes that require comprehensive management strategies. Traditional Chinese Medicine (TCM) offers a promising approach to preventing and treating these conditions, with a long history of use and a growing body of scientific evidence to support its effectiveness. TCM treatments, such as acupuncture, herbal remedies, and dietary interventions, may target various mechanisms underlying obesity and diabetes, including inflammation, oxidative stress, insulin resistance, and gut microbiota dysbiosis. However, further research is needed to elucidate the precise mechanisms of action and optimize the use of TCM to manage these disorders and ensure the safety and quality of TCM products. Given that most T2D is caused by obesity, it makes sense to favor treatment techniques that encourage weight loss. It is also necessary to consider the usage of specific ‘anti-obesity’ drugs to supplement an individual’s attempts to improve their lifestyle. The combination of obesity/diabetes medications and glucose-lowering agents, as well as the usage of some pharmaceuticals in any category for both purposes, blur the line between obesity and diabetes therapy. SGLT2i and GLP-1 RAs, for example, are already available glucose-lowering medicines that induce modest weight loss and are anticipated to play a larger role in diabetes care in the future, especially given the positive findings of their usage in recent cardiovascular outcome trials. Novel obesity-specific medications, on the other hand, offer potential in diabetes management, and, as a result, their use in diabetes treatment appears likely to increase over time.

## Author contributions

All authors contributed to the article and approved the submitted version.
